# Supplementary material for: Factors affecting care of elderly patients among nursing staff at the Ho teaching hospital in Ghana: Implications for geriatric care policy in Ghana
Source: PLoS One. 2022 Jun 23;17(6):e0268941. doi: 10.1371/journal.pone.0268941 (PMC9223345; doi:10.1371/journal.pone.0268941)
Supplement: Attachment — Submitted filename: Response to Reviewers.docx [file pone.0268941.s002.docx]

**Manuscript title:**

**A descriptive cross-sectional study of factors affecting care of elderly patients among nursing staff at the Ho teaching hospital: implications for geriatric care policy in Ghana**

**Short title:** **Factors affecting care of elderly patients among nurses.**

^*^Solomon Mohammed Salia^1^, **Peter Adatara^1^,** Agani Afaya^1,2^, Waliu Salisu Jawula^3^, Milipaak **Japiong**^1^, Abubakari Wuni^4^, Martin Amogre Ayanore^5^, Jacob Erwontaa Bangnidong^6^, Felix Hagan^1^, Dorcas Sam-Mensah^1^, **Robert Kaba Alhassan ^7^**

Dear editor,

We are happy for the opportunity to make inputs into our manuscript to the required journal’s standard for publication. We would like to thank the reviewers for the thorough review and insightful comments on our manuscript. The comments are very useful and we have responded to them to the best of our knowledge. We acknowledge that the comments have no doubt helped improve the quality of our manuscript.

We have therefore responded to the comments to our very best and have provided further details by showing point-by-point feedback on how each of the comments by reviewer # 2 was addressed. For easy identification of our responses, the reviewers’ comments have been repeated while the Authors’ responses appear in **BOLD** text in the main manuscript.

We also wish to notify you of the change in authorship. The change in authorship have been agreed by all authors to reflect their various contribution to the completion of the manuscript. **Peter Adatara** is placed as second author from the seventh position while **Robert Alhassan Kaba** is placed as the last author from the sixth position. We have therefore included a change to authorship form to reflect these changes.

Also, the author’s name “Japion” should be spelt as (**Japiong**).

Again, the institutional affiliation address of “Waliu Salisu Jawula” has been changed to his new institution’s address.

Additionally, the title of the manuscript has been modified to include the study setting as captured in the revised manuscript

All these changes have appeared on the revised manuscript.

.

**Reviewer’s Comments and Authors Responses**

Reviewer #1: This article is a descriptive cross sectional study regarding nurses’ positive attitudes towards elderly patients and the factors that influence attitudes. The topic is of great significance, given the increase of life expectancy, across the globe and the findings are of particular interest. Thank you for the opportunity to review this manuscript. The tables add greatly to the manuscript. However, the manuscript would be upgraded with a significant editing for writing and language quality. Findings are confirmatory of previous research; however, studies which indicated negative attitudes are not cited. A comparison among the different findings (positive VS negative attitudes) would be interesting. Strengths of the manuscript are the well-structured abstract, validity/reliability report and the tables. Weakness of the study is that literature needs to be updated and make a conceptual analysis of the terms that are used.

**Major issues**

**Reviewer’s Comment**
1. The scientific background is explained in the introduction section, though recent research is not cited. This manuscript would be benefited if more recent literature was cited. The originality of the study is supported, however gaps of the literature needs to be addressed. Furthermore, the writers mention the caring attitude, which is a very interesting topic, therefore needs to be developed and put more emphasis how is correlated with the study’s aim.

**Authors Response**

Authors acknowledged these important comments and have therefore revised the background accordingly. Please see the revisions on page 3-4 lines 123-170.

**Reviewer’s Comment**
2. Page 12: Ethical approval: How was nurse’s consent obtained? Verbally? Written?

**Authors Response**

A written informed consent was obtained from the nurses prior to the commencement of the study. This is included in the revised manuscript. Please see page 6 line 237-238.

**Reviewer’s Comment**
3. Page 13: Study variables: This paragraph needs better clarification. Maybe a revision would be helpful.

**Authors Response**

Study variables section of the manuscript was revised to include clarification. Please see page 7 line 307-308.

**Reviewer’s Comment**

4. Page 13: Data collection: This paragraph needs to be rephrased for better clarification. Where was the instrument development based? Also the use of reference is suggested, since a literature review was made.

**Authors Response**

The data collection instrument section of the manuscript was revised and the changes made in the revised manuscript. Please see the revisions on page 7-8 line 319-332.

**Reviewer’s Comment**
5. Page 13: Data collection procedure: How the missing data were handled?

**Authors Response**

Missing data were handled using the list-wise deletion of incomplete/missing entries before data analysis. Please see the revisions on page 8 lines 347-349.

**Reviewer’s Comment**
6. Page 14: Data analysis: This paragraph needs further development. How was Likert scale interpreted/calculated? Was it dichotomized? Also, it needs more details on percentages’ calculation.

**Authors Response**

The questionnaire was ranked on a five-point Likert scale with appropriate descriptions; thus; 1 (strongly disagree), 2 (disagree), 3 (neutral), 4 (agree) and 5 (strongly disagree). During the analysis, the Likert scale was recoded and dichotomized into two groups; Agree (strongly agree and agree) and Disagree (strongly disagree, disagree and neutral). Please see revision on page 8 line 324-326.

In the analysis of knowledge and attitude, nurses’ who obtained less than 50% of the total score were classified as having poor knowledge or negative attitude while those who scored more than 50% of the total score were classified as good knowledge or positive attitude. Please see the revisions on page 9 lines 370-373

**Reviewer’s Comment**
7. Page 16: Barriers and challenges: The authors should clarify this section to avoid confusion.

**Authors Response**

The authors have revised the manuscript and have agreed to use the word “barriers” and not challenges.

**Reviewer’s Comment**
8. Page 17: Factors that influence nurses’ attitude towards the care of elderly: This paragraph is quite interesting. The first sentence needs to be rephrased to enhance understanding.

**Authors Response**

The manuscript was revised to rephrase the sentence which added more clarity to the paragraph. Please see the revisions on Page 12 line 457-458.

**Reviewer’s Comment**
9. Discussion: Study objectives are clearly addressed but fail to connect findings with previous research in this area. The authors should rewrite the discussion to reference the relevant literature. More specifically:

a) Data description and the importance of the findings are the main strengths of the discussion section. However, the reference of the statistical analysis and a critical analysis of study’s outcome were absence.
b) “Caring attitude”, “empathy” and “compassion” should be developed conceptually. A clear connection with the topic of interests needs to be established.

c) Nurses’ negative attitudes towards elderly have been detected in several studies (Kada et al, 2009, Robinson et al, 2014, Evripidou et al, 2018). This manuscript would be promoted by including those studies and compare the findings.

d) Additionally, factors influencing attitudes needs further development. The article could be enriched by including studies (Elvish et al, 2014, Shinan-Altman et al, 2014, Evripidou et al, 2018) which have made a detailed report to factors that influence nurses’ attitudes.

**Authors Response**

The authors have revised the discussion section of the manuscript to address all the reviewer comments and have also made inputs based on reviewer suggestions. Please see the revisions on Page 16-19 line 481-628.

**Reviewer’s Comment**
10. The reference style that the journal uses is Vancouver. Please revise.
**Authors Response**

The manuscript was revised and the referencing style changed from APA to Vancouver referencing style including intext citations. Please see the revisions on Page 22-24.

**Minor issues**
11. Page 11: last paragraph, first sentence: needs reference.

**Authors Response**

This comment has been addressed in page 13 line 476.

**Reviewer’s Comment**
12. Study area: It is worth mentioning the reasons for the selection of the particular departments.

**Authors Response**
The study area section of the manuscript has been revised to include the reason for the choice of the wards for the study. Please see the revisions on Page 6 line 255-260.

**Reviewer’s Comment**

13. The validity and reliability paragraph is well structured. The use of reference would be an additional strength of this paragraph.

**Authors Response**

The validity and reliability section of the manuscript was revised to provide references that supported our findings on Cronbach’s Alpha Coefficient values. Please see the revisions on Page 8 line 358-363.

**Reviewer’s Comment**

14. Tables are clear and concise. Table 3 needs to be re-numbered. There are two tables numbered as “Table 4”.

**Authors Response**

The authors revised the manuscripts and the table numbers thus; table 3 and table 4 renumbered appropriately. Please see page 12 and 14.
