# Supplementary material for: Factors affecting care of elderly patients among nursing staff at the Ho teaching hospital in Ghana: Implications for geriatric care policy in Ghana
Source: PLoS One. 2022 Jun 23;17(6):e0268941. doi: 10.1371/journal.pone.0268941 (PMC9223345; doi:10.1371/journal.pone.0268941)
Supplement: Attachment — Submitted filename: Response to Reviewers.docx [file pone.0268941.s003.docx]

**Manuscript title:**

**A descriptive cross-sectional study on factors affecting care of elderly patients among nursing staff at the Ho teaching hospital: implications for geriatric care policy in Ghana**

**Short title:** **Factors affecting care of elderly patients among nurses.**

^*^Solomon Mohammed Salia^1^, **Peter Adatara^1^,** Agani Afaya^1,2^, Waliu Salisu Jawula^3^, Milipaak **Japiong**^1^, Abubakari Wuni^4^, Martin Amogre Ayanore^5^, Jacob Erwontaa Bangnidong^6^, Felix Hagan^1^, Dorcas Sam-Mensah^1^, **Robert Kaba Alhassan ^7^**

Dear editor,

We are happy for the opportunity to make inputs into our manuscript to the required journal’s standard for publication. We would like to thank the reviewers for the thorough review and insightful comments on our manuscript. The comments are very useful and we have responded to them to the best of our knowledge. We acknowledge that the comments have no doubt helped improve the quality of our manuscript.

We have therefore responded to the comments to our very best and have provided further details by showing point-by-point feedback on how each of the comments by reviewer # 2 was addressed. For easy identification of our responses, the reviewers’ comments have been repeated while the Authors’ responses appear in **BOLD** text in the main manuscript.

All these changes have appeared on the revised manuscript.

.

**Reviewer’s Comment**
A professional language editing would be beneficial. Some paragraphs on the introduction and the discussion needs to be re-written, so as to increase coherence.

**Authors Response**

Authors acknowledged these important comments and have therefore conducted proof reading on the whole manuscript. Some redundant sentences were removed and, in some cases, rephrased. Some of the rephrased sentences appeared in bold text. The authors also removed some of the references from the citations to prevent a case of “reference over-kill”

**Reviewer’s Comment**
The data collection instrument also needs further explanation.

**Authors Response**

The authors acknowledged this comment as very important. After a careful look at the description of the data collection instrument again all the authors came to a conclusion that the necessary ingredients were all present in its description. Therefore, we did not add further explanation to it.
