# Supplementary material for: Factors affecting care of elderly patients among nursing staff at the Ho teaching hospital in Ghana: Implications for geriatric care policy in Ghana
Source: PLoS One. 2022 Jun 23;17(6):e0268941. doi: 10.1371/journal.pone.0268941 (PMC9223345; doi:10.1371/journal.pone.0268941)
Supplement: Attachment — Submitted filename: Response to Reviewers.docx [file pone.0268941.s004.docx]

**Manuscript title:**

**Factors affecting care of elderly patients among nursing staff at the Ho teaching hospital in Ghana: implications for geriatric care policy in Ghana**

**Short title: Factors affecting care of elderly patients among nurses.**

^*^Solomon Mohammed Salia^1^, Peter Adatara^1^, Agani Afaya^1,2^, Waliu Salisu Jawula^3^, Milipaak Japiong^1^, Abubakari Wuni^4^, Martin Amogre Ayanore^5^, Jacob Erwontaa Bangnidong^6^, Felix Hagan^1^, Dorcas Sam-Mensah^1^, Robert Alhassan Kaba^7^

Dear editor,

We are happy for the opportunity to make inputs into our manuscript to the required journal’s standard for publication. We would like to thank the reviewers for the thorough review and insightful comments on our manuscript. The comments are very useful and we have responded to them to the best of our knowledge. We acknowledge that the comments have no doubt helped improve the quality of our manuscript.

We have therefore responded to the comments to our very best and have provided further details by showing point-by-point feedback on how each of the comments by reviewer # 3 was addressed.

The authors would also like to bring to your notice the need to delete a statement found in paragraph 5 page 3 & 4 in the “introduction section” of the manuscript which talks about undesirable attitudes of nursing staff towards the elder patient. However, in line 136-137, the authors found a statement which read **“Moreover, Elebiary and Abulazm (13) reported an overall positive attitude of nurses about aged care”.** We felt this should have been part of the positive attitudes section. The authors found this statement inappropriate to be in that paragraph and have therefore deleted in the submitted revised manuscript.

For easy identification of our responses, the reviewers’ comments have been repeated while the Authors’ responses appear in **BOLD** text in the main manuscript.

All these changes have appeared on the revised manuscript.

Please below our response to the review comments by reviewer # 3

**Reviewer’s comment**

A listing of the questions used to operationalize "good knowledge of the aging process", a "positive caring attitude", "good knowledge of geriatric care" and similar constructs is needed. Or at least cite some exemplary questionnaire items that were used. Otherwise it will not be clear to readers what "good" and "positive" consisted of, and hence will give the appearance of not being sufficiently empirical. This clarification could be done either in the methodology section of the text, or an appendix

**Authors Response**

The authors view this comment very useful and believe it will help the readers understand how good and poor knowledge and attitude as used this study meant. This will also add value to the construct of the questionnaire and study. To this, the authors included citations of the studies they modelled when the questionnaire was designed in the “data collection instrument section” of the methodology. Again, the authors have included relevant citations in the “analysis” section of the methodology where the knowledge and attitude classification occurred for further clarity. See in from page 6 line: 256-259 and page 7 line: 306-310.

**Reviewer’s comment**

Shorten/condense the article title to something more manageable such as... "Factors affecting care of elderly patients among nursing staff at the Ho teaching hospital in Ghana"

**Authors Response**

The authors find this comment very useful and have shortened the manuscript title to; Factors affecting care of elderly patients among nursing staff at the Ho teaching hospital in Ghana: implications for geriatric care policy in Ghana. See page 1 line 3-4.
